# Editorial: Frontiers in Brain-Based Therapeutic Interventions and Biomarker Research in Child and Adolescent Psychiatry

**DOI:** 10.3389/fpsyt.2016.00123

**Published:** 2016-07-05

**Authors:** Paul E. Croarkin, Stephanie H. Ameis

**Affiliations:** ^1^Noninvasive Brain Stimulation Program, Department of Psychiatry and Psychology, Mayo Clinic Depression Center, Mayo Clinic, Rochester, MN, USA; ^2^The Margaret and Wallace McCain Centre for Child, Youth and Family Mental Health, Temerty Centre for Therapeutic Brain Intervention, Slaight Centre for Youth in Transition, Campbell Family Mental Health Research Institute, Centre for Addiction and Mental Health, Toronto, ON, Canada; ^3^Centre for Brain & Mental Health, The Hospital for Sick Children, Toronto, ON, Canada; ^4^Child & Youth Mental Health Division, Department of Psychiatry, Faculty of Medicine, University of Toronto, Toronto, ON, Canada

**Keywords:** adolescent, biomarker, child, child and adolescent psychiatry, neuroimaging, non-invasive brain stimulation, pediatric

**The Editorial on the Research Topic**

**Frontiers in Brain-Based Therapeutic Interventions and Biomarker Research in Child and Adolescent Psychiatry**

Childhood psychiatric disorders present challenges given the heterogeneity of presentations, instability of phenotypes, and nascent understanding of neurodevelopment. Recent efforts, such as the National Institute of Mental Health Research Domain Criteria, aim to hone precision medicine approaches for psychiatric disorders (1). Elucidating the ontogeny of psychiatric illnesses and underlying neurobiology is a mandate for advancing modern clinical practice. Recent advances in neuroimaging, preclinical studies, genomics, and non-invasive brain stimulation may soon provide improved monitoring of development in health and disease. These tools also hold great promise for developing biological markers of illness that may be targeted through treatment innovation. This research topic surveys recent developmentally informed clinical neuroscience efforts focused on conditions that affect children and adolescents. Broadly, this includes studies focusing on neurodevelopmental disorders, eating disorders, mood disorders, and treatment innovations.

## Neurodevelopmental Disorders

Recent changes in descriptive diagnostic criteria, such as DSM-5 (2), aim to bridge basic science findings with clinical practice (3). Goldani et al. examine existing literature focused on putative biomarkers of autism spectrum disorder (ASD). Markers of mitochondrial function, oxidative stress, genetic clustering, and inflammation are promising approaches. However, at present, there is insufficient evidence to embed these markers into clinical practice (Goldani et al.). In other efforts to better understand neurobiology in ASD and related neurodevelopmental disorders, Baribeau and Anagnostou review neuroimaging correlates of ASD and schizophrenia. Volumetric changes, cortical thickness differences, and white matter changes in childhood onset schizophrenia (COS) appear to attenuate with age. Impaired local connectivity may also be coupled with amplified long-range connectivity in this condition. Neuroimaging findings in ASD collectively suggest an initial period of brain verdancy followed by dysmorphogenesis in adolescence. Furthermore in ASD, patterns of local hyper-connectivity are coupled with impaired long-range neural communication (Baribeau and Anagnostou). Nagamitsu et al. present recent dimensional work with brain single-photon emission computed tomography (SPECT) in participants with attention deficit hyperactivity disorder (ADHD). Children with ADHD, and higher scores on the child behavior checklist-dysregulation profile had significantly increased ^123^I-iomazenil biding in the posterior cingulate cortex. These data suggest that GABAergic inhibitory neurons are involved in the pathophysiology of ADHD (Nagamitsu et al.).

## Affective Disorders

Recent controversies surrounding the phenotyping of childhood mood disorders underscore the necessity of ongoing work focused on the neurobiological characterization of affective disorders during neurodevelopment (4). Henderson et al. examine white matter microorganization as assessed with diffusion tensor imaging (DTI) in adolescents with depression and healthy controls. Anhedonia and irritability were associated with unique neuroanatomical signatures. This promising early work suggests that prefrontal and limbic tracts are disrupted in depression and unique symptom presentations may have DTI signatures (Henderson et al.). Lee et al. examine brain function of healthy control participants, high-risk offspring, and youth with bipolar disorder by means of a meta-analytic approach. The authors postulated that greater activity in high-risk participants signifies potential compensatory mechanisms, whereas more widespread findings in bipolar patients signified chronic disease processes (Lee et al.). Finally, Walker et al. propose a compelling model for the mood disorder prodrome. These authors posit that early life stress, inflammation, and allosteric load are key contributors to disease burden, disease progression, and neuropathology (Walker et al.).

## Eating Disorders

Eating disorders are severely impairing psychiatric illnesses, with high mortality rates, and profound neurobiologic underpinnings (5). Herein, McAdams and Krawczyk describe findings from a study of patients with anorexia, patients with bulimia, and healthy controls. Participants were exposed to social attribution, social identity, and physical identity fMRI tasks. Consistently throughout each region of interest, average activation levels for bulimic participants were greater than the group of patients with anorexia, but less than healthy participants. The authors concluded that patients with eating disorders could have a similar biological substrate in terms of social functioning, yet a distinctive functional characterization is a plausible pursuit for future work (McAdams and Krawczyk). Nagamitsu et al. also present intriguing work focused on developing SPECT biomarkers to guide the treatment of children with anorexia nervosa. In children with anorexia nervosa, decreased ^123^I-iomazenil binding in the anterior cingulate and left parietal cortices was associated with a suboptimal response to treatment. Successful weight restoration was associated with increased relative binding of ^123^I-iomazenil in the posterior cingulate and occipital cortices (Nagamitsu et al.).

## Treatment Innovation in Children and Youth

Transcranial magnetic stimulation (TMS) is a powerful therapeutic and neurophysiological probe. Neurocognitive outcomes are key both in terms of safety and for intervention development, as they may serve as optimal clinical outcome measures in youth (6, 7). Wall et al. report on a study in which eighteen depressed adolescents received 30 sessions of 10-Hz rTMS, applied to the left dorsolateral prefrontal cortex. Participants demonstrated improvements in delayed verbal recall and memory. Furthermore, there were no decrements in other neurocognitive dimensions (Wall et al.). Desarkar et al. postulate that imbalances in excitatory and inhibitory neurotransmission could underlie aberrant neuroplasticity in ASD. At the receptor level, this may involve excessive NMDA and deficient GABA-mediated neurotransmission. Interventions with high frequency rTMS may have a role in stabilizing dysregulated neuroplasticity in ASD (Desarkar et al.).

In conclusion, the synthesis of neuroscience with child and adolescent psychiatry is yielding important discoveries and new directions for treatment innovation. However, we have yet to make the discoveries necessary to bring neuroscience research into the clinical realm through specific biomarker discovery that could pave the way for precision medicine where biomarkers are profiled in the clinic and individualized treatments are selected to optimize neurodevelopmental trajectories, mitigate the long-term effects of psychiatric illness, and maximize functioning for individuals. Novel research tools, innovative study designs that go beyond the case–control model, longitudinal research that identifies developmental trajectories within heterogeneous conditions, and large-scale studies with the power to detect small effects are likely the next frontier in research focused on advancing our understanding of neurobiological underpinnings and developing biologically informed treatments for children and youth with mental illness.

## Author Contributions

All authors listed have made substantial, direct, and intellectual contribution to the work and approved it for publication.

## Conflict of Interest Statement

Dr. PC has received in-kind support for research (supplies and genotyping) from Assurex Health. He has received in-kind support for equipment from Neuronetics. He has received travel support and research support (investigator-initiated trial and serves as a site primary investigator for a multicenter study) from Neuronetics. He has received research support from Pfizer (investigator-initiated grant). Dr. SA has no financial conflicts of interest to declare.
